# Dynamics of Phosphorus and Biostimulants on Agro-Morphology, Yield, and Essential Oil Profile of German Chamomile (*Matricaria chamomilla* L.) Under Acidic Soil Conditions of the Western Himalaya

**DOI:** 10.3389/fpls.2022.917388

**Published:** 2022-07-25

**Authors:** Shalika Rathore, Rakesh Kumar

**Affiliations:** ^1^Agrotechnology Division, CSIR-Institute of Himalayan Bioresource Technology, Palampur, India; ^2^Academy of Scientific and Innovative Research (AcSIR), Ghaziabad, India

**Keywords:** German chamomile, triple superphosphate, amino acid, humic acid, bisabolol oxide

## Abstract

German chamomile (*Matricaria chamomilla* L.) is a promising and easy to cultivate crop under suitable nutrient supply conditions, but acidic soils of Indian western Himalayas limit the availability of phosphorus to the plant and reduce flower production. Thus, a field experiment was conducted for two consecutive seasons (2018–2019 and 2019–2020) to study the effect of phosphorus dynamics and biostimulant application on the agro-morphological traits, essential oil (EO) yield, and chemical constituents of German chamomile in the mid hills of the western Himalayan region. The experiment consisted of 12 treatments, four phosphorus fertilizer levels (0, 30, 60 and 90 kg ha^−1^) and three biostimulants levels (control, amino acid at 5 mL L^−1^, and humic acid at 10 mL L^−1^). The experiment was replicated three times in a factorial complete randomized block design (FRBD). Agro-morphological and yield characteristics were significantly higher in phosphorus at 90 kg ha^−1^ and humic acid application compared to the control. Dry flower and EO yield was 17.87 and 26.76% higher with the 90 kg ha^−1^ phosphorus application while 2.45 and 5.79% higher in humic acid at 10 mL L^−1^ compared to the control. The EO constituents *viz*., chamazulene was 12.04 and 8.85% higher in phosphorus at 90 kg ha^−1^ and humic acid at 10 mL L^−1^ application compared to the control. On the other hand, α-bisabolol oxide B and α-bisabolol oxide A were decreased with increase in phosphorus application. This study presents novel facts, elucidation, and explanation for farmers and industrialists to produce German chamomile in acidic soils by integrating biostimulants with phosphorus fertilization and getting maximum yield and quality EO.

## Introduction

Medicinal and aromatic plants (MAPs) have served as the root of traditional medicine around the world since time immemorial (Gurib-Fakim, 2006). They are used extensively in herbal medicines and other sectors such as food, cosmetics, and perfumery (Zouaoui et al., 2020). German chamomile (*Matricaria chamomilla* L.) is globally one of the important MAPs (family: *Asteraceae*) used in a diverse range of food, cosmetics, and perfumery sectors and has been used traditionally as a herbal medicine for thousands of years in Greece, Egypt, and Rome (Mann and Staba, 2002), but the major confront in the agriculture system is to boost crop production with a sustainable approach for short- and long term-goal achievements (Fallah et al., 2020).

It is more regrettable that long-term continuous disproportionate fertilizer application has reduced soil organic matter and quality, thus declining agricultural production and enhancing environmental pollution (Guo et al., 2010). Hence, all the aforesaid issues have become significant concerns at present (Chaudhry et al., 2009). The nutrient elements, *viz*., nitrogen (N) and phosphorus (P) are particularly significant in MAPs, because they are involved in the structures of the precursors in EO, and in enzymes and energy-carrying molecules such as ATP (Dehsheikh et al., 2020). Thus, the use of plant nutrients cannot be neglected, but the source of these can be modified from chemical to organic origin (derived from plant and animal wastes) to achieve the sustainable agriculture goal (Fallah et al., 2018).

The soil pH values in the mid-hill Himalayan region range from 4 to 6 (acidic) because of high rainfall and highly weathered soils. In acidic soils, P becomes unavailable to plants as it gets fixed in the soil by sesquioxides, eventually leading to P deficiency. Thus, P becomes the most limiting nutrient in the crop production system (Johan et al., 2021). The reduced level of available P can adversely affect plant height, lateral plant spread, flower number, flower size, biomass, pollen production, etc. (Jiang et al., 2017). P is next to N in plant macronutrient supply, and P fertilizers are primary sources that can be applied to increase P availability to plant roots, thus enhancing crop growth, development, and production. In flowering plants, P has a major role in the flower development and reproduction stages (Kumar et al., 2006). With German chamomile being a flowering plant, it is imperative to study the effect of P on morphological and EO quality traits. The application of a certain amount and type of P fertilizer has a straight influence on the flowering, weight of flowers, and EO yield of German chamomile (Omidbeygi, 1995).

Biostimulants are natural preparations (organic substances and/or microorganisms) that increase plant nutrient utilization efficiency and abiotic stress tolerance and improve crops' quality without causing adverse side effects (Rouphael et al., 2018). They include humic and fulvic acids, salicylic acid, phenols, amino acids, proteins, enzymes, and micronutrients (Jardin Du, 2015). Humic acid is a hormone-like substance used for plant nutrition and improves nutrient absorption and plant growth (El-Gohary et al., 2019). It also has a positive effect on cell membrane functions by biosynthesis of nucleic acids, ion absorption, and respiration (Yang et al., 2004). Humic acid improves plant hormones and responsiveness, because it inhibits indole acetic acid oxidase activity, leading to increased IAA hormone activity and encouraging plant growth (El-Gohary et al., 2019). Other than this, among natural biostimulants, amino acid-based biostimulants with high content of free amino acids are prepared by enzymatic hydrolysis. Moreover, the increase in customers' consciousness regarding healthy products favors the enhancement and significance of organic production, and farmers are also keen on using natural stimulants to improve crop quality (Drobek et al., 2019). Effects of the foliar application of humic acid (De Hita et al., 2020) and amino acids (Vassilev, 2016) were studied on some morphological, physiological, and biochemical characteristics of cucumber (*Cucumis sativus* L.) and sunflower (*Helianthus annuus* L.). There is a dearth of information on German chamomile in response to P and biostimulants from the high precipitation region of the western Himalaya hence an attempt has been made to study the effect of P and biostimulants on German chamomile.

## Materials and Methods

### Experiment Site

**Figure 1:**
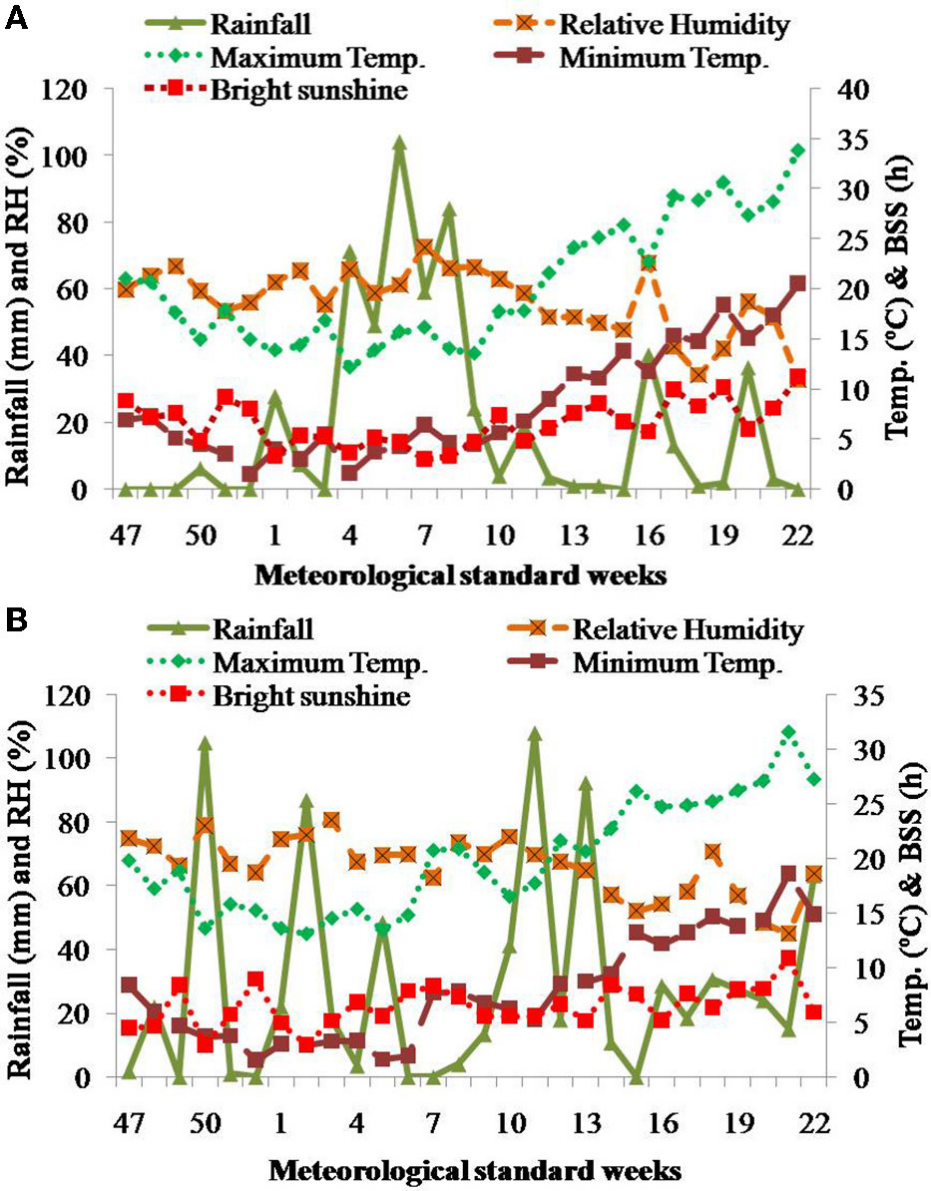
Mean weather conditions during the crop growth seasons **(A)** 2018–2019 (Upper graph) and **(B)** 2019–2020 (Lower graph) at the experimental cultivation site in Palampur, HP, India. BSS, bright sunshine hour; RH, relative humidity.

A field experiment was conducted for 2 repeated years, during 2018–2020 in the experimental farm of CSIR-IHBT (Council of Scientific and Industrial Research- Institute of Himalayan Bioresource Technology), Palampur, Himachal Pradesh (HP), India situated 1,325 m above mean sea level (amsl) altitude (32°11′39"N latitude and 76°56′51"E longitude). The climate of the region was subtropical, and the soil was characterized as clayey loam with acidic pH (5.34 ± 0.06), low in available P, i.e., 5.3 ± 0.35 P_2_O_5_ kg ha^−1^, low organic carbon (0.28 ± 0.03%), medium in available nitrogen (234.24 ± 2.01 kg ha^−1^), and high in available potassium (292.09 ± 3.44 K_2_O kg ha^−1^). Weather parameters, *viz.*, minimum and maximum temperature (°C), relative humidity (RH%), and average bright sunshine (BSS) hours during crop growth season, were acquired from “Crop weather outlook”, an agro-meteorological advisory (Anonymous, 2020) and are illustrated in Figure 1. The maximum temperature (34°C) was recorded in May and the minimum temperature (2°C) in December. During 2018–2019, mean RH was maximum (73%) in February and minimum (33%) in May. The received total rainfall in German chamomile growth season during 2018–2019 was 557 mm, with the maximum in February and the lowest in December, while the received average daily BSS was 7 h. The maximum (32°C) and minimum (2°C) temperatures were recorded in May and February, respectively. Mean RH was maximum (81%) in January and minimum (45%) in May. The received total rainfall in German chamomile growth season during 2019–2020 was 803 mm, with the maximum in March and the lowest in February, while the received average daily BSS was 6 h. The total precipitation of the region during crop growth duration was lesser during the first growth year while it was higher in the second growth year. Moreover, the precipitation during the first growth year was higher in February (320 mm), i.e., the time period when plants were in the vegetative stage; while during the second growth year, the precipitation was higher from March to April (317 mm), i.e., the period when plants were blooming.

### Factors and Experiment Details

The design used was a factorial randomized block design (FRBD) with three replications under field conditions. The experiment consists of 12 treatments with two factors, i.e., four phosphorus (P_2_O_5_) fertilizer levels, *viz*., P1 0 kg ha^−1^ (control), P2 30 kg ha^−1^, P3 60 kg ha^−1^, and P4 90 kg ha^−1^, and three biostimulant levels, B1: control (distilled water), B2: amino acid at 5 mL L^−1^ and B3: humic acid at 10 mL L^−1^. Treatment combinations of different levels of treatments factors were 12 allocated randomly in each block replicated thrice in the prepared field. The biostimulants are commercially available in the market under the brand name Amino Booster G (the amino acid solution) and V-Hume (the humic acid solution), the total phosphorus content in the amino acid and the humic acid was 0.03 and 0.12%, respectively; these were purchased for experiment execution. Well-decomposed farmyard manure (FYM at 15 t ha^−1^) was applied 2 months before seed sowing; for better uniformity and distribution of plants in the field, the seeds were mixed with sand. The seed sowing of accession I (α-bisabolol oxide A-rich chemotype) was conducted in equidistant rows with a spacing of 40 cm in each experimental plot (size: 4 × 2.25 m). During both crop-growing years, the recommended dose of fertilizer (1/3 nitrogen and complete potassium) was given through urea (N 46%) and muriate of potash (K_2_O) as pre-sowing fertilization; the remaining N was applied during the crop elongation and flower initiation stages in equal amounts. The P fertilizer used in the experiment was triple super phosphate (TSP) (CaH_4_P_2_O_8_) and mixed in the soil before seed sowing. Biostimulants were applied by foliar application by dissolving in distilled water, and concentrations of 5 and 10 mL L^−1^ of the amino acid and humic acid, respectively, were prepared. The concentrations of the biostimulants and distilled water (control) were then sprayed before the flower bud formation stage on the treated plants and the control plants, respectively. As the flowering of German chamomile is not synchronous, spraying of the treatments was carried out fortnightly (after the first spray) from the end of the spring season (March) to the summer season (May) during both years. Weeding and irrigation procedures were carried out as per crop requirement.

### Plant Growth, Pigment, and Yield Determination

Morphological features, *viz*., plant height (in cm) and numbers of branches were recorded at crop harvest (full flowering) by placing a quadrant (25 cm^2^) in two sites in the sampling rows of each plot. Growth-contributing attributes such as photosynthetic photon flux density (PPFD, in μmol^−1^s^−1^ m^2^) and leaf area index (LAI) were measured with an LI-191R line quantum sensor (measures PAR) and Handheld Laser LAI Meter CI-110/120 (CID, Bio-Science, United States), respectively. The major flowering flush started from mid-April to May; flower plucking was carried out with a 2-week interval. The number of flowers was evaluated by placing a quadrant (25 cm^2^) in two sites in the core region row of the plot, and flower drying (up to constant weight) was conducted at room temperature, i.e., 20 to 25°C during April and 25 to 30°C during May; then, cumulative dry flower yield was calculated. Fresh leaf samples (1 g) were taken, washed with distilled water, homogenized with acetone (80%), and centrifuged at 5,000 rpm for 5 min, and the absorbance (OD) of the supernatant was recorded at λ = 663, 646, and 470 nm with a spectrophotometer (T90 + UV spectrophotometer; PG instruments Ltd.) for chlorophyll (Chl a and Chl b) and carotenoid determination. Chlorophyll (a and b) and carotenoid (in mg g^−1^) content was determined (Lichtenthaler and Buschmann, 2001).

Chl a = 12.21 OD_663_-2.81 OD_646_,

Chl b = 20.13 OD_646_-5.03 OD_663_, and

Carotenoids = (1,000 OD_470_-3.27 Chl a−104 Chl b)/229.

### Identification and Determination of Essential Oil Components

Fresh flowers were harvested from the net plot area of each treatment, dried at room temperature, and hydrodistilled in triplicates for 4 h in a Clevenger-type apparatus (European Pharmacopoeia, 2007). EO content was calculated as volume (in mL) of EO obtained per weight (in g) of flowers, and the EO was dried with anhydrous sodium sulfate (Na_2_SO_4_). The EO was then stored at 4°C in a glass container before analysis. EO yield was calculated by multiplying the EO content with the specific gravity (0.92) of EO and total dry flower yield of all the harvests. GC and GC/MS analyses were executed in triplicates with a flame ionization detector (FID) on a Shimadzu GC 2010 gas chromatograph and QP2010 (Shimadzu Corp., Tokyo, Japan) fitted with an AOC 5000 auto-injector. The auto-injector consisted of a 30-m long ZB-5 MS capillary column with a 0.25-mm i.d. and a 0.25-μm thick film (SGE International, Ringwood, Australia). Ten μL EO was dissolved in 2 mL of dichloromethane and auto-injected in split mode with 2 μL volume. N (carrier gas) was used at a 1.05 mL min^−1^ flow rate; the temperature of the oven was maintained at 70°C for 3 min and subsequently risen to 220°C for 5 min at a rate of 4°C min^−1^. The maintained temperature of the injector was 220 °C while that of the detector was 250°C. The settings of temperature lineup, injection volume, and carrier gas utilized to execute GC and GC/MS were similar to the detailed procedure for *Rosmarinus officinalis* L. in Rathore et al. (2022). To identify components from areas of GC peaks, a series of hydrocarbons was used for retention index (RI) determination without the use of any correction factor. EO components were identified by matching the experimental RIs with the RIs reported in the literature (Adams, 2017; Rathore and Kumar, 2021). Moreover, the identification was also conducted by comparing and matching the minimum mass spectral fragmentation pattern of the components with the NIST library (Stein, 2005).

### Statistical Analysis

Once the morphological and chemical composition evaluation was completed, data were analyzed with the analysis of variance (ANOVA) technique for factorial RBD. Least significant testing was completed by Fisher's least significant difference (LSD) test and regarded as statistically significant at *p* = 0.05. Analysis of variance for effect of cropping year, phosphorus and biostimulant on growth and yield traits of *M. chamomilla* was also studied (Table 1). EO constituents were subjected to multivariate principal component analysis to determine the expression of treatments in EO constituents with software (PCA software PAST 3).

**Table 1:** Analysis of variance for effect of cropping year, phosphorus and biostimulant on growth and yield traits of *M. chamomilla*.

**Sources of variations**	**Df**	**Plant height**	**Number of branches**	**Number of flowers**	**Flower diameter (cm)**	**Flower yield (t ha^**−1**^)**	**Essential oil yield (kg ha^**−1**^)**
Year	1	NS	NS	**	**	**	**
Phosphorus	3	**	**	**	**	**	**
Biostimulant	2	**	**	**	**	**	**
Year × Phosphorus	3	NS	**	NS	NS	**	**
Y × Biostimulant	2	NS	NS	NS	NS	*	*
Phosphorus × Biostimulant	6	**	NS	NS	**	NS	NS
Year × Phosphorus × Biostimulant	6	NS	NS	NS	NS	NS	NS

* and ** represent significant relationship at p = 0.05 and p = 0.01, respectively. ns, not–significant; Df, degree of freedom.

## Results

### Plant Growth and Pigment Investigation

**Figure 2:**
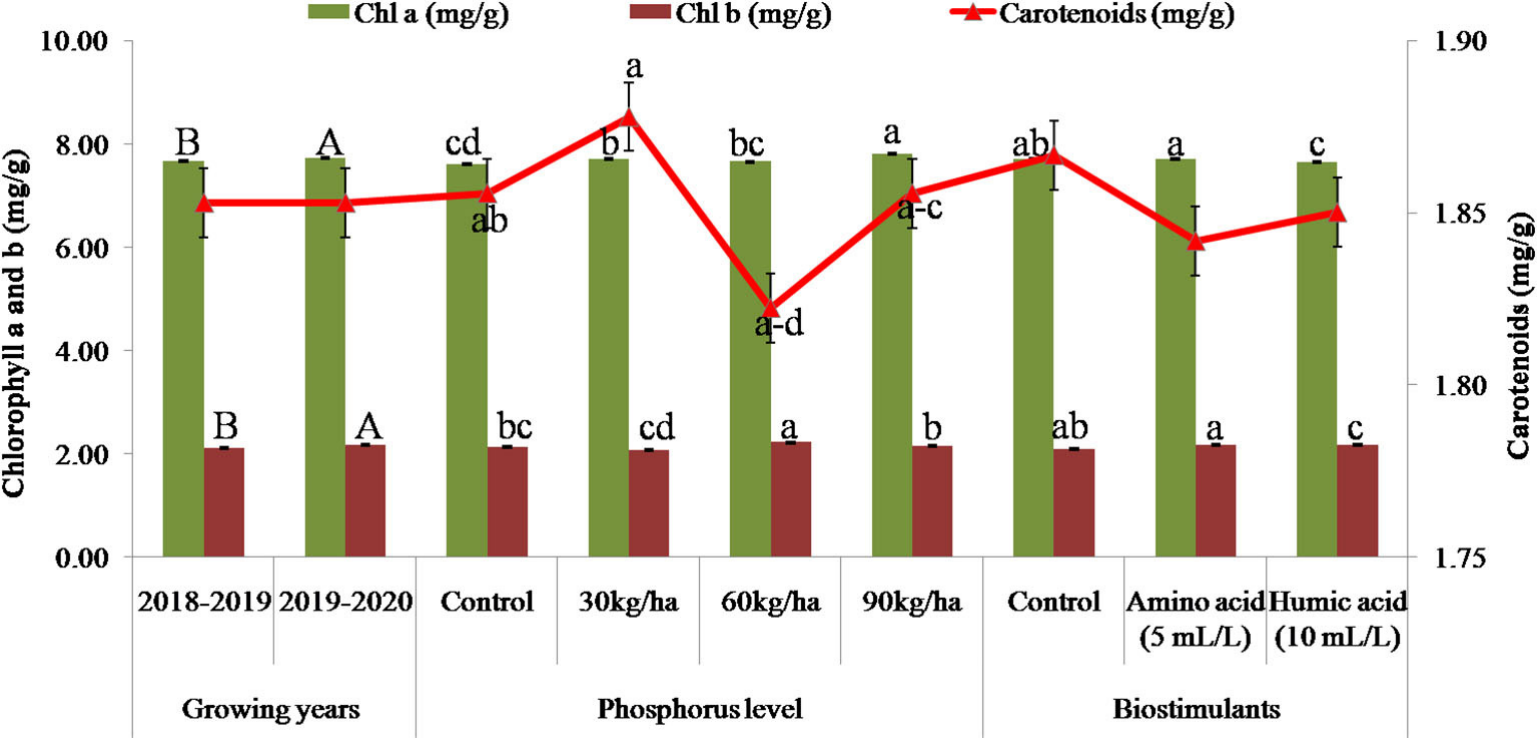
Growing years, phosphorus and biostimulant application affected the chlorophyll and carotenoids (mg g^−1^) concentration at harvest in *M. chamomilla*.

At harvest, plant growth attributes, *viz*., plant height and number of branches per plant, were not significantly affected by growth years, but photosynthetically active radiation (PAR) (575.81 μ mol s^−1^ m^−1^) and LAI (3.1) were significantly higher during 2018–2019 than during 2019–2020 (Table 2). Significantly higher concentrations of Chl a and Chl b were recorded during 2019–2020; while carotenoids were not significantly affected by the growing years (Figure 2). Similarly, significantly higher plant height (83.8 cm), PAR (583.78 μ mol s^−1^ m^−1^), and LAI (3.24) were recorded in P at 90 kg ha^−1^ compared with the control, while the number of branches was significantly higher in P at 60 kg ha^−1^ compared with the control but remained at par with 90 kg ha^−1^ P application (Table 2). Likewise, Chl a was significantly higher in P at 90 kg ha^−1^ as compared to the control, while Chl b and carotenoids were significantly higher in the P at 60 and 30 kg ha^−1^ applications as compared to the control (Figure 2). Significantly higher plant height (83.24 cm) and number of branches per plant (23.17) were recorded in the humic acid application compared to the control. PAR (μ mol s^−1^ m^−1^) accumulation was measured as difference in PAR above plant canopy and below plant canopy; usually much flourished plant canopies contribute to more PAR accumulation by plants. The PAR (578.08 μ mol s^−1^ m^−1^) accumulation by the plants was significantly higher in the amino acid compared to the other biostimulant applications, and LAI (3.03) was significantly higher in the humic acid application compared to control the but remained at par with the amino acid application (Table 2). The concentration of Chl a (7.7) and carotenoids (1.87) was significantly higher in the control than in other biostimulant treatments, while Chl a was significantly higher in the amino acid application (Figure 2).

**Table 2:** Growing years, phosphorus and biostimulant application influences plant growth and yield contributing attributes in *M. chamomilla* at harvest.

**Treatment**	**Plant height (cm)**	**Number of branches per plant**	**PAR**	**LAI**
**Growing years**				
2018–2019	75.46	22.36	575.81	3.10
2019–2020	74.62	22.25	571.81	2.92
SEm (±)	0.78	0.15	1.21	0.01
LSD (*p* = 0.05)	NS	NS	3.43	0.03
**Phosphorus levels**				
Control	67.90	21.04	559.89	2.84
30 kg ha^−1^	70.74	21.78	574.22	2.97
60 kg ha^−1^	77.66	23.27	577.33	2.98
90 kg ha^−1^	83.87	23.13	583.78	3.24
SEm (±)	1.10	0.22	1.70	0.02
LSD (*p* = 0.05)	3.13	0.62	4.85	0.04
**Biostimulants**				
Control (0 mL L^−1^)	69.79	21.43	570.00	2.98
Amino acid (5 mL L^−1^)	72.10	22.32	578.08	3.03
Humic acid (10 mL L^−1^)	83.24	23.17	573.33	3.03
SEm (±)	0.95	0.19	1.48	0.01
LSD (*p* = 0.05)	2.71	0.53	4.20	0.04

*SEm ±, standard error mean; LSD, least significant difference (p = 0.05); NS, not significant; PAR, photosynthetically active radiations; LAI, leaf area index*.

**Figure 3:**
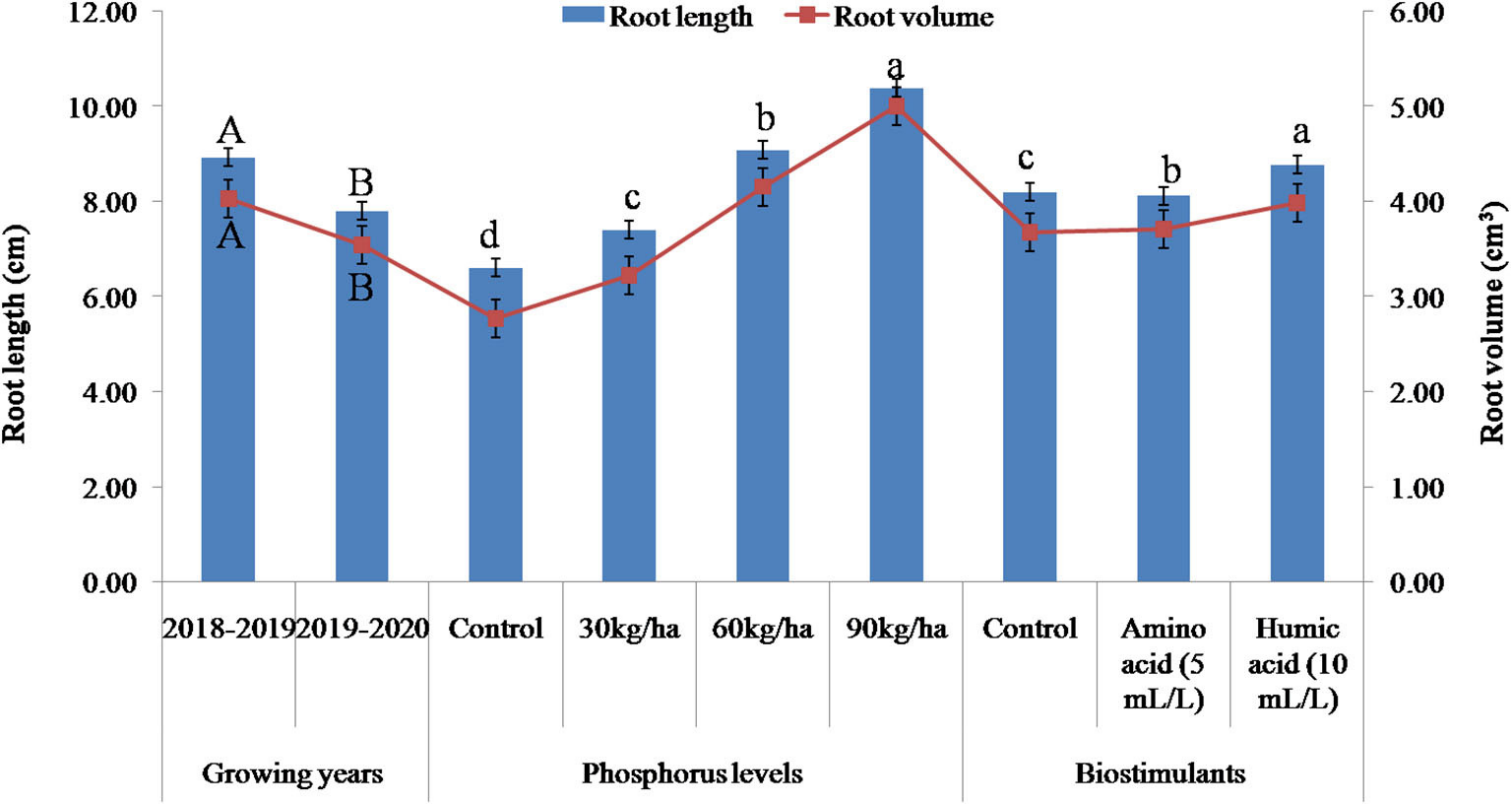
Growing years and phosphorus and biostimulant application affected root length (in cm) and root volume (in cm^3^) at harvest in *M. chamomilla*.

The application of biostimulants significantly affected PAR and LAI. Significantly higher PAR (578.08 μ mol s^−1^ m^−1^) was recorded in the amino acid than in the humic acid and control, while LAI (3.03) was significantly higher in the humic acid application than in the control but remained at par with the amino acid application (Table 2). In the crop harvest stage, root length and root volume were significantly affected by the growing years and P and biostimulant applications (Figure 3). Significantly higher root length (8.93 cm) and root volume (4.03 cm^3^) were recorded in 2018–2019 than in 2019–2020. Likewise, P at 90 kg ha^−1^ and humic acid recorded significantly higher root length (10.38 cm) and root volume (5 cm^3^) than the control (Figure 3).

### Yield Attributes, Essential Oil (EO) Content, and Yield

At crop harvest, the number of flowers per plant (71.56), dry flower weight (6.8 g plant^−1^), flower diameter (2.29 cm), disc floret height (89.69 mm), dry flower yield (2.46 t ha^−1^), EO content (0.79%), EO yield (17.59 kg ha^−1^) were significantly higher during 2019–2020, while the number of ray florets was not significantly affected by the crop-growing years (Table 3). The number of flowers per plant (75.24), flower diameter (2.3 cm), disc floret height (92.33 mm), and dry flower yield (2.63 t ha^−1^), EO yield (19.17 kg ha^−1^) were significantly higher in P at 90 kg ha^−1^ than in the control. Similarly, dry flower weight (7.05 g plant^−1^) and EO content (0.81%) were significantly higher in P at 90 kg ha^−1^ but remained at par with the 60 kg ha^−1^ P application. Likewise, the number of ray florets was also significantly higher in P at 90 kg ha^−1^ than in the control but remained at par with the 60 and 30 kg ha^−1^ P applications. The application of biostimulants significantly affected the yield attributes. The foliar application of humic acid recorded a significantly higher number of flowers (71.3), dry flower weight (6.72 g plant^−1^), and EO yield (17.25 kg ha^−1^) than the amino acid and control. Similarly, dry flower yield and EO yield were also significantly higher in humic acid than in the control but remained at par with the amino acid application, while flower diameter was significantly higher in the amino acid but remained at par with the humic acid application (Table 3).

**Table 3:** Growing years, phosphorus and biostimulant application influences yield attributes and yield in *M. chamomilla* at crop harvest.

**Treatment**	**Number of flowers**	**Dry flower weight (g plant^**−1**^)**	**Flower diameter (cm)**	**Number of ray florets per flower**	**Disc floret height (mm) per flower**	**Dry flower yield (t ha^**−1**^)**	**E. oil content (%)**	**E. oil yield (kg ha^**−1**^)**
**Growing years**								
2018–2019	67.63	6.26	2.12	22.94	88.31	2.38	0.74	15.96
2019–2020	71.56	6.80	2.29	22.72	89.69	2.46	0.79	17.59
SEm (±)	0.40	0.03	0.01	0.19	0.18	0.00	0.00	0.07
LSD (*p* = 0.05)	1.15	0.10	0.02	NS	0.52	0.01	0.01	0.21
**Phosphorus levels**								
Control	62.49	5.85	2.09	21.56	82.22	2.16	0.72	14.04
30 kg ha^−1^	67.02	6.30	2.19	23.00	89.89	2.34	0.74	15.63
60 kg ha^−1^	73.62	6.93	2.25	23.11	91.56	2.54	0.80	18.23
90 kg ha^−1^	75.24	7.05	2.30	23.67	92.33	2.63	0.81	19.17
SEm (±)	0.57	0.05	0.01	0.26	0.26	0.01	0.00	0.10
LSD (*p* = 0.05)	1.63	0.14	0.03	0.75	0.74	0.02	0.01	0.29
**Biostimulants**								
Control (0 mL L^−1^)	67.82	6.36	2.17	22.46	89.17	2.38	0.75	16.25
Amino acid (5 mL L^−1^)	69.66	6.52	2.24	22.96	88.67	2.43	0.77	16.81
Humic acid (10 mL L^−1^)	71.30	6.72	2.21	23.08	89.17	2.44	0.78	17.25
SEm (±)	0.50	0.04	0.01	0.23	0.23	0.00	0.00	0.09
LSD (*p* = 0.05)	1.41	0.12	0.03	NS	NS	0.01	0.01	0.25

*SEm ±, standard error mean; LSD, least significant difference (p = 0.05); NS, not significant*.

### Regression and Correlation Analysis

**Figure 4:**
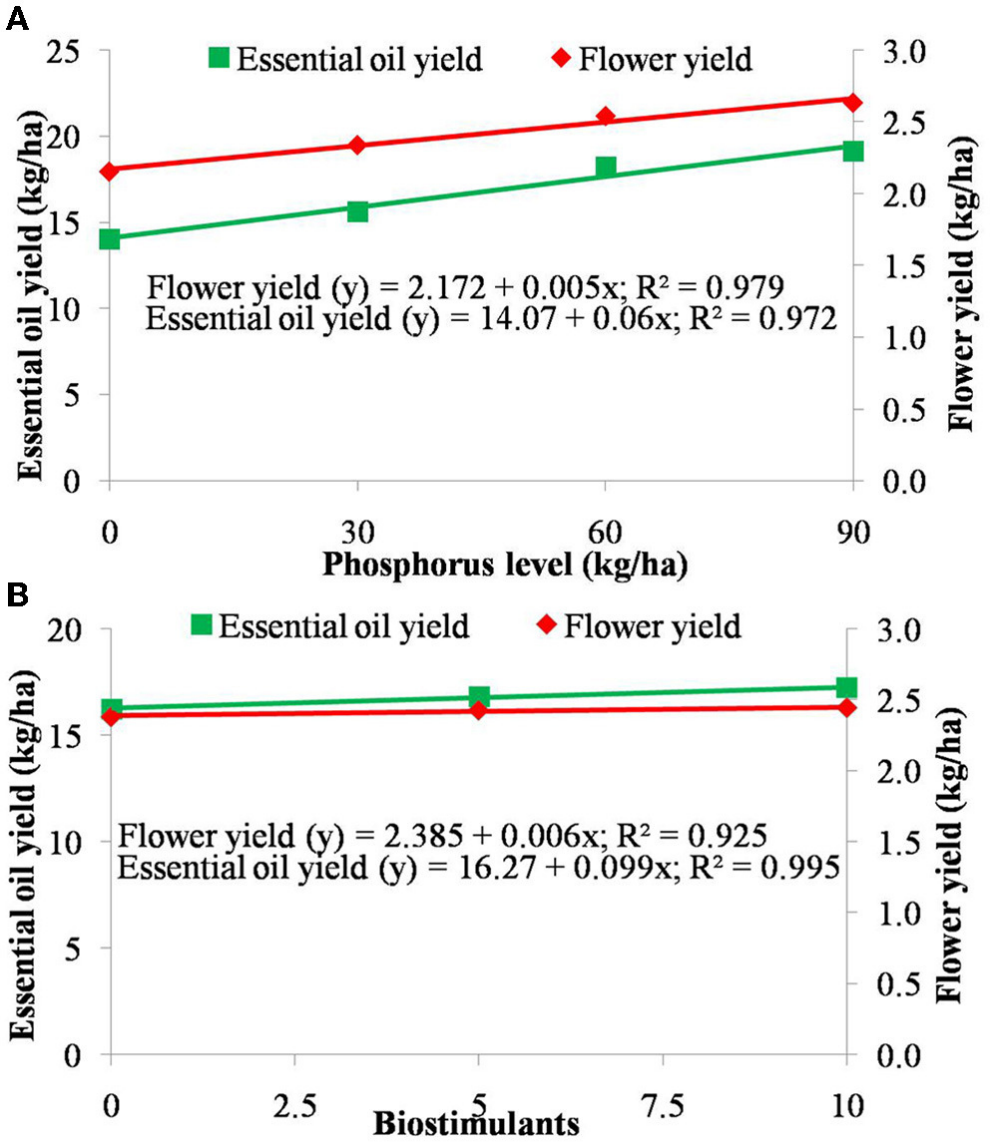
Regression equation between independent variable, **(A)** levels of phosphorus (upper graph) and **(B)** biostimulant and dependent variables (lower graph) i.e., total flower yield (in kg ha^−1^) and essential oil yield (in kg ha^−1^). The levels of phosphorus and biostimulants are represented in the primary *X* axis. Total essential oil yield and flower yield are presented in the primary *Y* axis and secondary *Y* axis, respectively.

Regression equations were illustrated among the independent variables, i.e., P and biostimulant applications, and dependent variables, i.e., flower yield and EO yield (Figure 4). The flower and EO yields in German chamomile increased with increase in P application, and the highest yield was observed at 90 kg P ha^−1^. The application of P developed a strong linear relationship with flower yield and EO yield using equations y = 2.172 + 0.005x, (*r*^2^ = 0.979) and y = 14.07 + 0.06x, (*r*^2^ = 0.972) at *P* = 0.01), respectively (Figure 4A). The application of P from 0 to 30 kg ha^−1^ and from 30 to 60 kg ha^−1^ recorded a considerable increase in flower yield and EO yield, but this increase was much slighter from 60 to 90 kg ha^−1^ P application. Similarly, flower yield and EO yield increased with the application of the biostimulants and developed a strong relationship, i.e. y = 2.385 + 0.006x, (*r*^2^ = 0.925) and y = 16.27 + 0.099x, (*r*^2^ = 0.995) respectively (Figure 4B).

**Figure 5:**
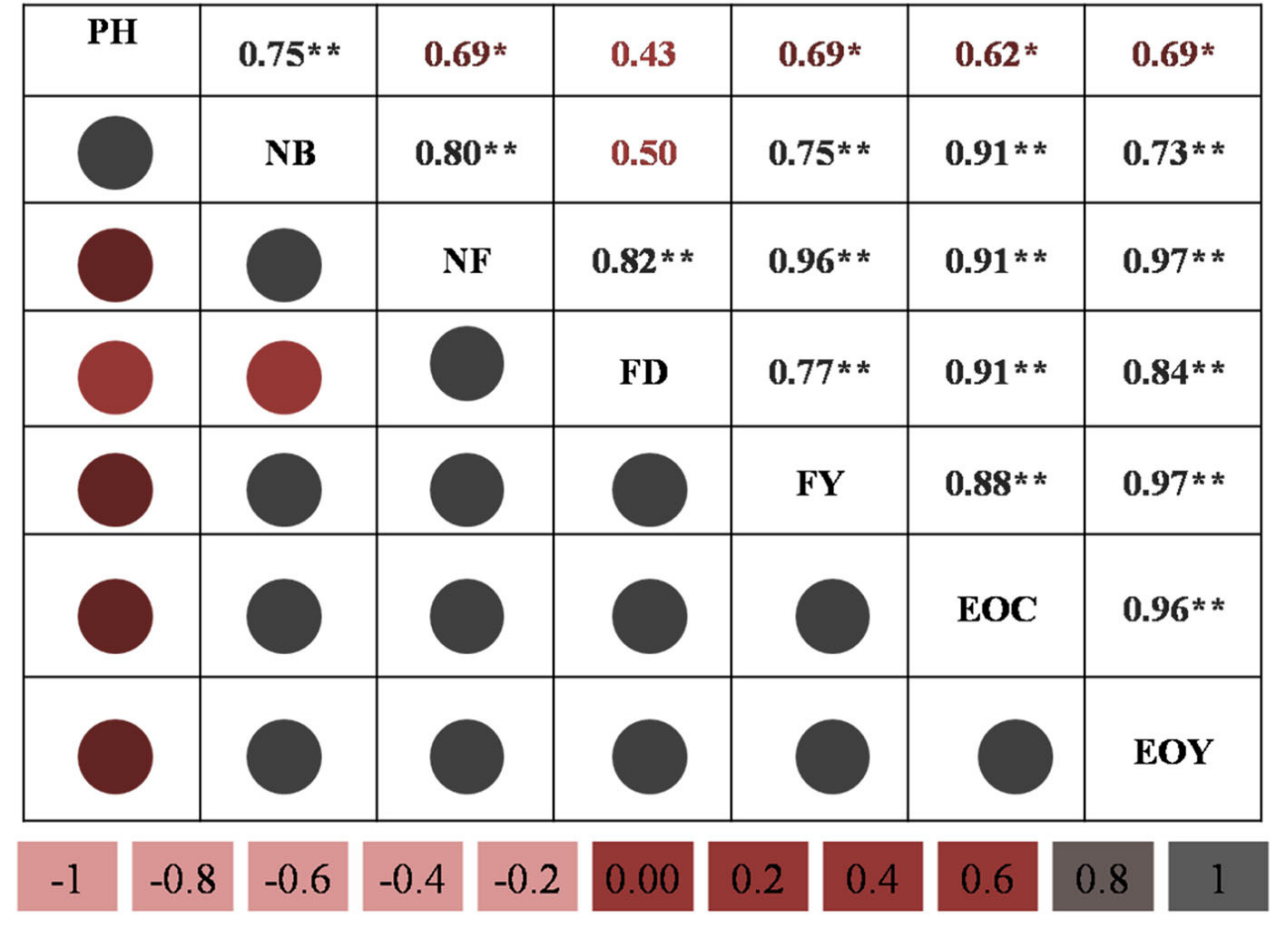
Correlation analysis of growth and yield parameters. PH, plant height; NB, number of branches; NF, number of flowers; FD, flower diameter (in cm); FY, dry flower yield (in t ha^−1^); EOC, essential oil content (in %); EOY: essential oil yield. The mean values of the 2-year pooled data of the corresponding treatments are used; * and ** indicate that the corresponding values are significant at *p* = 0.05 and *p* = 0.01, respectively.

The correlation matrix recorded a significant (*P* = 0.01) correlation of EO yield with number of branches (*r* = 0.73), number of flowers (*r* = 0.97), flower diameter (*r* = 0.84), flower yield (in t ha^−1^) (*r* = 0.97), EO content (*r* = 0.96) and a significant correlation with plant height (cm) (*r* = 0.69) at *P* = 0.05 significance level (Figure 5). Similarly, EO content recorded a significant (*P* = 0.01) correlation with number of branches (*r* = 0.91), number of flowers (*r* = 0.91), flower diameter (cm) (*r* = 0.91), and flower yield (t ha^−1^) (*r* = 0.88), and showed a significant (*P* = 0.05) correlation with plant height (*r* = 0.62). Also, flower yield showed a significant (*P* = 0.01) correlation with number of branches (*r* = 0.75), number of flowers (*r* = 0.96), and flower diameter (cm) (*r* = 0.77). Moreover, a significant (*P* = 0.01) correlation was observed between flower diameter and number of flowers (*r* = 0.82), while a positive correlation was observed with number of branches (*r* = 0.5) and plant height (*r* = 0.43). Number of flowers showed a significant correlation with number of branches (*r* = 0.8), and number of branches further showed a significant correlation with plant height (*r* = 0.75) at the *P* = 0.01 significance level.

### Determination of Essential Oil Components

The major EO constituents (more than 5% area) in EO were (Z)-β-farnesene, germacrecene D, α-bisabolol oxide B, bisabolone oxide A, α-bisabolol oxide A, and en-in-dicycloether, while artemisia ketone and chamazulene were reported as minor constituents (>2% area contribution) (Table 4). The concentration of (Z)-β-farnesene, germacrecene D, and en-in-dicycloether was significantly higher in 2019–2020, while that of α-bisabolol oxide B, bisabolone oxide A, chamazulene, and α-bisabolol oxide A was significantly higher in 2018–2019. Likewise, the application of biostimulants recorded a significantly higher concentration of (Z)-β-farnesene and germacrecene D with the 30 kg ha^−1^ P application, while α-bisabolol oxide B and α-bisabolol oxide A were significantly higher in the control. On the other hand, chamazulene and en-in-dicycloether were significantly higher with the 90 kg ha^−1^ P application compared to the other P levels. Similarly, the application of biostimulants significantly affected the EO constituents and recorded significantly higher (Z)-β-farnesene, germacrecene D, chamazulene, α-bisabolol oxide A, and en-in-dicycloether in the humic acid application than the amino acid and control. On the other hand, α-bisabolol oxide B and bisabolone oxide A were significantly higher in the control and amino acid applications than the other treatments while later remaining at par with the control (Table 4).

**Table 4:** Growing years, phosphorus and biostimulant application influences essential oil composition in *M. chamomilla*.

**Treatment**	**Ocimene < (E)-β->**	**Artemisia ketone**	**Camphor**	**β-farnesene**	**Germacrene D**	**Bicyclogermacrene**	**α-farnesene**	**Spathulenol**	**Epi-α-Cadinol**	**α-bisabolol oxide B**	**Bisabolone oxide A**	**Chamazulene**	**α-bisabolol oxide A**	**En-in-dicycloether**
**Growing years**														
2018–2019	0.18	2.64	0.79	5.87	0.79	1.56	0.91	0.97	0.95	13.91	12.47	2.76	42.99	3.19
2019–2020	0.94	2.95	0.52	8.22	8.22	0.96	0.50	0.45	0.62	10.02	11.33	2.47	40.59	11.90
SEm (±)	0.18	0.06	0.02	0.08	0.04	0.02	0.03	0.04	0.04	0.08	0.09	0.04	0.14	0.09
LSD (*p* = 0.05)	0.50	0.16	0.06	0.24	0.11	0.05	0.07	0.12	0.12	0.21	0.25	0.10	0.39	0.26
**Phosphorus levels**														
Control	0.41	2.47	0.54	6.72	4.90	1.05	0.70	0.54	0.77	13.26	11.60	2.41	44.77	6.06
30 kg ha^−1^	1.10	3.64	0.59	8.71	5.16	1.14	0.85	0.64	0.98	11.72	11.15	2.67	39.98	6.36
60 kg ha^−1^	0.46	2.51	0.85	5.81	3.90	1.74	0.74	0.72	0.76	11.45	12.75	2.66	41.40	8.39
90 kg ha^−1^	0.28	2.58	0.65	6.94	4.07	1.13	0.53	0.95	0.63	11.42	12.12	2.74	41.01	9.36
SEm(±)	0.25	0.08	0.03	0.12	0.05	0.03	0.04	0.06	0.06	0.11	0.13	0.05	0.19	0.13
LSD (*p* = 0.05)	NS	0.22	0.09	0.33	0.15	0.07	0.10	0.17	0.17	0.30	0.36	0.15	0.55	0.37
**Biostimulants**														
Control (0 mL L^−1^)	0.46	3.28	0.59	7.20	4.42	1.14	0.59	0.71	0.81	12.67	12.23	2.47	41.42	6.42
Amino acid (5 mL L^−1^)	0.44	2.68	0.85	6.61	4.37	1.60	0.76	0.70	0.78	11.49	12.37	2.67	41.67	7.87
Humic acid (10 mL L^−1^)	0.79	2.43	0.53	7.32	4.73	1.05	0.76	0.72	0.76	11.73	11.10	2.71	42.29	8.35
SEm(±)	0.22	0.07	0.03	0.10	0.05	0.02	0.03	0.05	0.05	0.09	0.11	0.04	0.17	0.11
LSD (*p* = 0.05)	NS	0.19	0.08	0.29	0.13	0.06	0.09	NS	NS	0.26	0.31	0.13	0.47	0.32

*SEm ±, standard error mean; LSD, least significant difference (p = 0.05); NS, not significant*.

### Principal Component Analysis (PCA)

**Figure 6:**
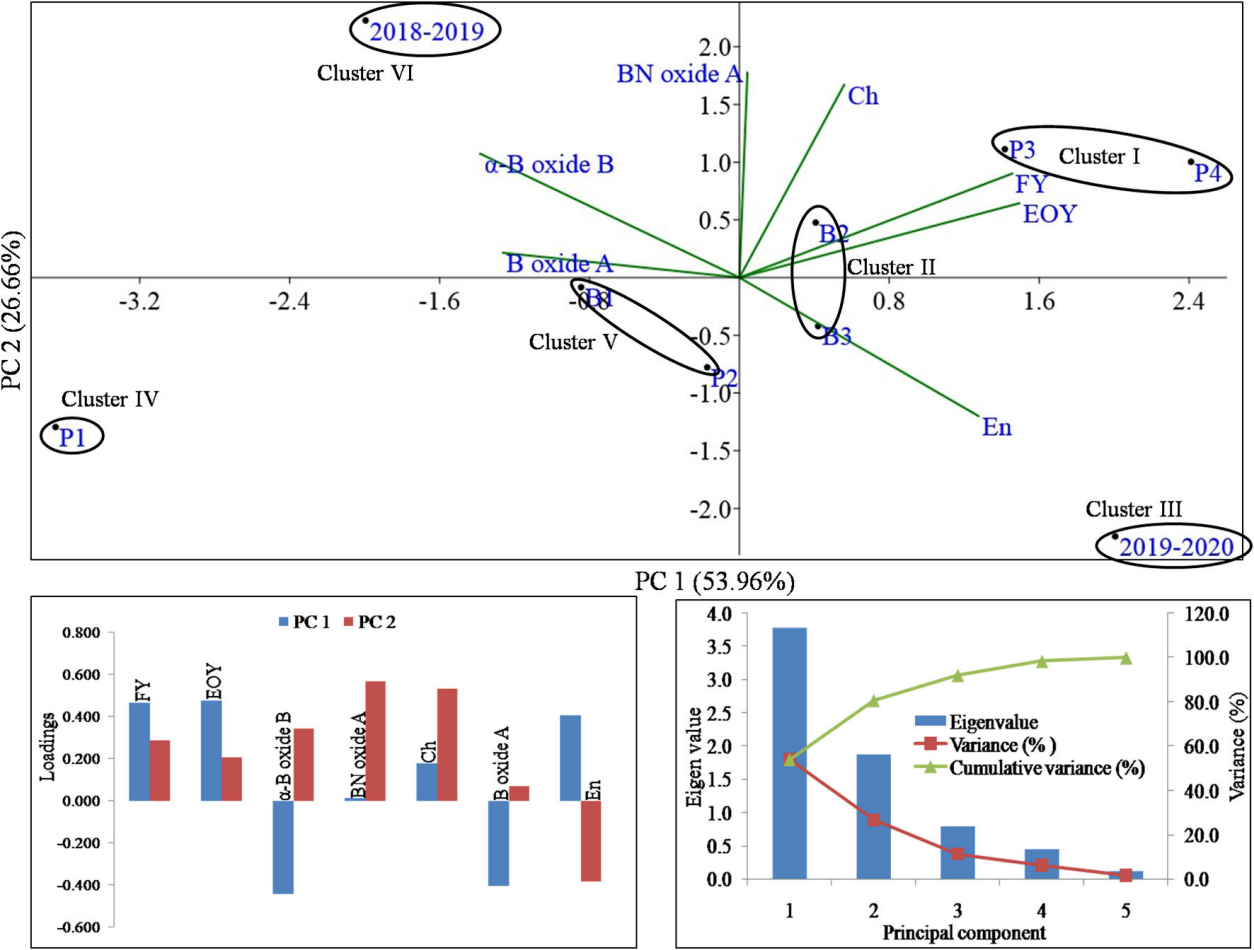
The multivariate analyses of mean value of growth parameters, yield and major compounds of essential oil were conducted through principal component analysis. PC1 and PC2 jointly explained the variations of 80.62%. The loading values of variables and treatment combinations are presented as vectors in the space of the PCA. The eigenvalues and loading scores of the variables with PC1 and PC2 are presented in the bottom left and right corner, respectively. FY, dry flower yield (t ha^−1^); EOY, essential oil yield (Kg ha^−1^); α-B oxide B, α-bisabolol oxide B; BN oxide A, bisabolone oxide A; Ch, chamazulene; B oxide A, bisabolol oxide A; En, en-in-dicycloether.

The dependent variables such as flower yield (t ha^−1^) and EO yield (kg ha^−1^) and major EO constituents, *viz*., α-bisabolol oxide B, bisabolone oxide A, chamazulene, α-bisabolol oxide A, and en-in-dicycloether were subjected to principal component (PC) analysis to discover the relationship between growing years and the P and biostimulant applications (Figure 6). The PC analysis revealed that 80.62% of the total variations were explained by PC1 and PC2. Among all the variables, flower yield, EO yield, bisabolone oxide A, chamazulene, and en-in-dicycloether showed a positive association, while α-bisabolol oxide B and bisabolol oxide A showed a negative association in PC1. Similarly, in PC2, flower yield, EO yield, α-bisabolol oxide B, bisabolone oxide A, chamazulene, and bisabolol oxide A showed a positive association, while only en-in-dicycloether showed a negative association. The analysis through PC separated the P3, P4 (phosphorus levels), and B2 (amino acid application) treatments in PC1 and PC2 by showing a positive contribution in PCs, flower yield, EO yield, bisabolone oxide A, and chamazulene. The findings of the present study showed that the first three PCs were extremely informative, with eigen values of 3.8, 1.9, and 0.8 and thus contributed about 92% of the overall variance of the dependent variables. The observed score plot could be divided into six distinct clusters (Figure 6). Cluster I included treatments, *viz*., P3 and P4; cluster II included B2 and B3; cluster V included B1 and P2; clusters III, IV, and VI corresponded to 2019–2020, P1, and 2018–2019 treatments, respectively. Cluster I included higher ranges of flower yield (2.54–2.63 t ha^−1^), EO yield (18.23–19.17 kg ha ^−1^), and en-in-dicycloether (8.39–9.36%) in P3 and P4 phosphorus levels. Cluster II comprised the lowest bisabolone oxide A (11.1–12.37%) and intermediate ranges of flower yield (2.43–2.44 t ha^−1^) and EO yield (16.81–17.25 kg ha^−1^) in B2 and B3 biostimulant applications. Additionally, cluster II included the 11.49–11.73, 2.67–2.71, 41.67–42.29, and 7.87–8.35 ranges of α-bisabolol oxide B, chamazulene, bisabolol oxide A, and en-in-dicycloether, respectively. Cluster III included a single treatment level, i.e., the 2019–2020 growing year of the crop, which consisted of the lowest α-bisabolol oxide B (10.02%) and bisabolone oxide A (11.33%) range and the highest en-in-dicycloether (11.9%) concentration. Similarly, cluster IV again comprised of a single treatment level, i.e., P1 (no phosphorus application) with the lowest flower yield (2.16 t ha^−1^), EO yield (14.04 kg ha^−1^), and chamazulene (2.41%) and the highest bisabolol oxide A (44.77%) and intermediate range of en-in-dicycloether (6.06%) concentration. Cluster V included B1 and P2 levels of the biostimulant and P applications; flower yield, bisabolone oxide A, and bisabolol oxide A had the lowest range, i.e., 2.34–2.38 t ha^−1^, 11.15–12.23%, and 39.98–41.42% in cluster V. Cluster VI included a single treatment level, i.e., the 2018–2019 growing year of the crop that consisted of the highest ranges of α-bisabolol oxide B (13.91 %) and chamazulene (2.76%), and the lowest range of flower yield (2.38 t ha^−1^) and en-in-dicycloether (3.19%) was observed. In the end, a significant disparity in flower yield and EO yield was recorded in the different growing years and P and biostimulant applications under the open field conditions of western Himalayan foothills (Table 5).

**Table 5:** Clusters variability in dependent variables in *M. chamomilla* affected by growing years, phosphorus and biostimulant application.

**Variables**	**Cluster I**	**Cluster II**	**Cluster III**	**Cluster IV**	**Cluster V**	**Cluster VI**
Flower yield	2.54–2.63	2.43–2.44	2.46	2.16	2.34–2.38	2.38
Essential oil yield	18.23–19.17	16.81–17.25	17.59	14.04	15.63–16.25	15.96
α-bisabolol oxide B	11.42–11.45	11.49–11.73	10.02	13.26	11.72–12.67	13.91
Bisabolone oxide A	12.12–12.75	11.10–12.37	11.33	11.60	11.15–12.23	12.47
Chamazulene	2.66–2.74	2.67–2.71	2.47	2.41	2.47–2.67	2.76
Bisabolol oxide A	41.01–41.40	41.67–42.29	40.59	44.77	39.98–41.42	42.99
En-in-dicycloether	8.39–9.36	7.87–8.35	11.90	6.06	6.36–6.42	3.19

## Discussion

### Plant Growth and Pigment Investigation

The use and integration of plant biostimulants with fertilizers are increasing in traditional agriculture systems with an aim to enhance crop production. PAR accumulation, LAI, root length, and root volume were significantly affected by crop growth years, and variations can be attributed to diverse climatic conditions during crop growth years. The PAR at harvest was higher during the first growing year, which might be because of lesser precipitation during harvest months, i.e., end of March to mid-May on meteorological standard weeks (MSWs) 13 to 20 (Figure 1A) during the first growth year, while precipitation was higher at harvesting time in the second growing year on MSWs 13 to 20 (Figure 1B). Similarly, during the first crop-growing year, the higher LAI can be synchronized with lesser precipitation and higher solar radiation in terms of BSS, while higher precipitation and lesser BSS in the second crop growing year contributed to lesser LAI (Figure 1). Similar findings with higher PAR and LAI were reported in lower cloud cover and higher solar radiation in forest crop species of the Amazon forest (Li et al., 2018). The higher root attributes in the first growing year might be attributed to lesser average precipitation in the crop growing period, because enhanced root growth can be inferred as an adaptive strategy to deal with lower water availability due to lesser precipitation during growing season by increasing absorptive root surface (Metcalfe et al., 2008). Similar to the present findings, the production of roots in *Artemisia barrelieri* Besser increased with a 30% rainfall reduction (Padilla et al., 2015). In contrast to the present findings, precipitation has no significant effect on root length and biomass allocation in *Erodium oxyrhynchum* M.Bieb. (Chen et al., 2019), while Cheng et al. (2006) reported an increase in root length as moisture in soil profile increased. The application of P at 90 kg ha^−1^ remained at par with 60 kg P ha^−1^ and recorded significantly higher plant height, number of branches, PAR, and LAI. This may be because of the fact that the soil of the experimental plot was acidic, and that the amount of P in the soil was very low, hence making it unavailable for root uptake and contributed to low plant height and number of branches in the control plots compared with the P-applied plots (Zheng, 2010). The findings are in accordance with Sonmez (2018), who reported higher plant height in P application than control in anise (*Pimpinella anisum* L.). Erbas et al. (2017) also recorded significantly higher plant growth with the application of 100 kg P ha^−1^ in lavandin. On the contrary, the P application did not significantly affect plant height and number of branches in rose geranium (*Pelargonium graveolens* L.) (Sedibe and Allemann, 2012). Similar to the present findings, P application increased the chlorophyll content in basil (Ramezani et al., 2009) and African marigold (Rathore et al., 1985) and the LAI (Maurya, 1989) in coriander genotypes. Similarly, the application of monopotassium phosphate at 0 to 3 g L^−1^ in *Rosa multiflora* Thunb. increased leaf chlorophyll content, root dry weight, total root length, fine root length, and surface area (Ma et al., 2021). P application increased root length and root volume, which corroborates the finding in wheat, where increased availability of P due to application of biochar increased root length by 1.4–1.8 times (Song et al., 2020). Likewise, the application of humic acid (75 mg kg^−1^) and P fertilizer (120 mg P_2_O_5_ kg^−1^ air-dried soil) in corn recorded higher root volume and other root attributes compared with the control (Purwanto et al., 2021).

A significant influence of biostimulants on agromorphological characteristics was observed, which is comparable to previous studies on lavandin (Erbas et al., 2017), garden thyme (*Thymus vulgaris* L.) (Kwiatkowski et al., 2020), and German chamomile (Mazrou et al., 2021). Similar to the present findings, increased plant height and number of branches in Dutch fennel (*Foeniculum vulgare* Mill.) (Mohamed, 2020) and increased photosynthetic pigments and LAI in basil (Amer et al., 2021) by foliar application of humic acid were observed. The beneficial influence of applying biostimulants on chlorophyll was due to their capacity to provide components, *viz*., betaines, phytohormones, polymers, and nutrients; thus, their synergetic action enhances endogenous cytokinins synthesis, which produced a protective effect on chloroplast (Zavaleta-Mancera et al., 2007) and, accordingly, affected the chlorophyll content.

### Yield Attributes and Essential Oil Content and Yield

The crop growth years influenced the yield attributes, which were significantly higher in the second growing year. This might be because of better accumulation of photosynthetically active radiation, which was correlated to improved growth in the second growth year and increased yield attributes. The yield attributes, *viz*., number of flowers, dry weight of flower, and flower diameter, were higher in the second crop growth year because of favorable prevailing weather conditions with reference to precipitation. The precipitation was higher during the second year between March to April, (Figure 1B) when plants were in the full flowering stages compared to first year when there was a considerable decrease in rainfall during the same period (Figure 1A), which reduced flowering attributes. The favorable precipitation condition in the 2nd year contributed positively to plant yield attributes, as the roots of German chamomile are shallow and capable of taking water from soil when precipitation is higher. At the same time, during the dry spell of the 1st growing year, there was less water absorption that eventually decreased the plant yield attributes. Similar to the present findings, higher rainfall contributed to more flower yield in chamomile in a typical Mediterranean climate with hot, dry summers and cold, humid winters (Guo et al., 2010). The yield attributes, *viz*., number of flowers per plant, flower diameter, disc floret height, dry flower yield, and EO yield, were significantly higher with P at 90 kg ha^−1^ that with the control. The higher yield attributes with P application were due to P's involvement in the floral structure development (Ma et al., 2001) of German chamomile, and similar improvement in floral characteristics was reported in lavandin (*Lavandula* × *intermedia* Emeric ex Loisel.) (Erbas et al., 2017), *Calendula officinalis* (Steward and Lovett-Doust, 2003), *Rosa multiflora* (Ma et al., 2021), and *Pimpinella anisum* (Meena et al., 2015). The highest dry flower and EO yields were produced with 90 kg ha^−1^ of P application (Table 3), because P influences several metabolic measures such as photosynthesis, chlorophyll content, carbohydrate, protein, and oil syntheses, thus contributing in EO yield increase (Erbas et al., 2017). Corroborating the current findings, P application increased the yield attributes, *viz*., seeds number per umbel, 1,000-seed weight, seed yield, and EO content in anise (*Pimpinella anisum* L.) (Sonmez, 2018), and EO yield in German chamomile with 150 kg ha^−1^ of phosphate fertilizer (TSP) with zinc application (Jeshni et al., 2015). On the contrary, the application of higher doses of P fertilizer (300 kg ha^−1^) showed a negative influence on German chamomile yield because the higher P application induced Zn deficiency in soil, thus creating an imbalance of nutrient supply (Jeshni et al., 2015). Moreover, the application of other nutrient fertilizers like N (Emongor and Chweya, 1992; Guo et al., 2010) also affected the performance of German chamomile. The application of NPK at 100:60:40 kg ha^−1^ recorded the highest growth and yield attributes of German chamomile (Gandomi et al., 2021). The application of NPK at 100:60:40 kg ha^−1^ recorded the highest growth and yield attributes of German chamomile (Upadhyay et al., 2016). Likewise, the application of micronutrients, i.e., zinc fertilizer (ZnSO_4_H_2_O) at 30 kg ha^−1^ and irrigation at 50% of field capacity, improved the EO yield and EO components of German chamomile (Jeshni et al., 2015). Additionally, the application of N fertilizer and the cultivation of legume stubble catch crops improved the performance of German chamomile. The cultivation of Dukat (*Anethum graveolens* L.) decreases fertilizer rate from 90 to 60 kg N ha^−1^ without decreasing anthodium yield. EO yield was highest after applying 60 kg N ha^−1^ (Andrzejewska and Woropaj-Janczak, 2014). In the present findings, biostimulant application significantly increased yield attributes, i.e., number of flowers, dry flower weight, and EO yield. Previous studies on humic acid influence were related to increased nutrient absorption and translocation, which facilitated the stimulation of plasma membrane H^+^-ATPases by transferring released free energy by ATP hydrolysis, thus generating electrochemical potential through the membrane and supplied nutrients (Jardin Du, 2015). Additionally, the ATPase membrane pump causes cell wall loosening and elongation, and expansion of cells, resulting in increased growth and chlorophyll content in plants (Abdelgawad et al., 2020). Moreover, humic acid is also known to induce modifications in gene expression and metabolites (primary and secondary) of plants (Baia, D. C., et al., 2020), which are involved in a variety of physiological processes (such as photosynthesis, metabolism, and Krebs cycle) (Carletti et al., 2018; Sofi et al., 2018). In ajowan, the application of humic acid with a chemical fertilizer recorded the highest EO content and main chemical constituents including thymol and *p*-cymene content (Chiyaneh et al., 2022). The increase in EO yield in German chamomile by the humic acid application may be due to stimulation of metabolic reactions and some enzymatic responses which are accountable for biosynthesis of EO and its constituents (Burbott and Loomis, 1969). Additionally, humic acid is a hormone-like substance and increases the levels of other internal plant hormones, *viz*., auxin, cytokinin, and gibberellin, which stimulate the division and elongation of plants cells (Abdel-Mawgoud et al., 2007) and improve plant growth (Abdel-Mawgoud et al., 2007). Applying supplementary biostimulants through amino acids facilitates the defense mechanisms in plants, prevents water loss, stimulates photosynthesis, determines metabolic processes' pace and path, and regulates internal enzymes and hormones (Kocira et al., 2015), which might have contributed to the improved characteristics of German chamomile compared to the control.

### Determination of Essential Oil Components

The crop growing years also showed a significant effect on EO constituents where (Z)-β-farnesene, germacrecene D, and en-in-dicycloether were higher in 2019–2020, while α- bisabolol oxide B, bisabolone oxide A, chamazulene, and α-bisabolol oxide A were higher in 2018–2019. In the present findings, α-bisabolol oxide A was the major EO constituent, followed by α-bisabolol oxide B and (Z)-β-farnesene in both growing years, which was in accordance with previous reports on German chamomile (Sharafzadeh and Alizadeh, 2011). During both years, some quantitative and qualitative differences in some components (α-bisabolol oxide A, α-bisabolol oxide B, and (Z)-β-farnesene) were observed, but the components did not lose their main characteristic, that is, richness. Even the concentrations of the three main components significantly differed according to variations in the weather parameters of both years but were close to each other. Similar to the present findings, a wide range of variability was observed in quality traits of fennel during growing years, as the studied traits might be influenced by environmental conditions (Lal, 2008). The EO production in aromatic crops may be influenced in a positive or negative manner by the amount and type of fertilizers (Ramezani et al., 2009; Said-Al Ahl et al., 2016). Similarly, P fertilization affected the composition of EO differently in lavender (Chrysargyris et al., 2016) and fennel (*Foeniculum vulgare* Mill.) (Kapoor et al., 2004), but there was no effect on sage (*Salvia officinalis* L.) (Rioba et al., 2015) and sweet basil (Chimura et al., 1993). In the present study, the major EO constituents were (Z)-β-farnesene, germacrecene D, α-bisabolol oxide B, bisabolone oxide A, α-bisabolol oxide A, and en-in-dicycloether, while artemisia ketone and chamazulene were reported as minor constituents. Earlier studies had also reported a similar EO profile of German chamomile with bisabolol oxide A as a major EO constituent (Orav et al., 2010; Can et al., 2012).

The EO composition of German chamomile in the present findings was in accordance with the standard ISO 19332:2008, which described α-bisabolol oxide A as 35–50% and chamazulene as 2–5% compound concentration in the EO supply. In aromatic crops, P is the primary macronutrient of plants and has an imperative function in the synthesis and assimilation of EO, where farnesyl pyrophosphate (FPP) is compressed to geranyle diphosphate (GPP) and linalool diphosphate, which are the precursor compounds of EO production (Sangwan et al., 2001). In the present study, the application of P at 90 kg ha^−1^ recorded significantly higher chamazulene and en-in-dicycloether, which is similar to the finding of Mikhak et al. (2017). On the other hand, chamazulene content was higher in the control plots (no phosphorus) but lower in the P treatments (Ubessi et al., 2021). Similar to the present findings, higher α- bisabolol oxide B and α-bisabolol oxide A content in control was also reported by Ubessi et al. (2021), while increase in α- bisabolol oxide B and α-bisabolol oxide A content was recorded with increase in P applications (Karami et al., 2006; Jeshni et al., 2015). According to the European Pharmacopoeia, there are two types of EO, one is rich in bisabolol oxides (between 29 and 81%) and the other in α-bisabolol (between 10 and 65%). Similarly, the EO constituents, *viz*., (Z)-β-farnesene, germacrecene D, chamazulene, α-bisabolol oxide A, and en-in-dicycloether were significantly higher in the humic acid application, while α-bisabolol oxide B and bisabolone oxide A were significantly higher in the control and amino acid applications. Comparable to the present findings, earlier studies also recorded variations in the chemical constituent profile of chamazulene and bisabolol contents with application of biostimulants, *viz*., salicylic acid (Ghasemi et al., 2016; Rathore and Kumar, 2021), in German chamomile. Moreover, the use of certain biostimulants such as humic acid, glycyrrhizic acids, salicylic acid, and their nanocomplexes in basil (*O. basilicum* L.) influences the biochemical attributes (Amer et al., 2021). These biostimulants may change the secondary metabolites' pathway, affect the plastid and chlorophyll levels and tolerance to stress conditions, and ultimately result in manipulation of the quantity and quality of EO (Ahmed, A. M., 2014). Humic acid improves nutrient accessibility for plants and is essential for the development and division of glandular trichomes, secretory ducts, and EO channels (Salehi et al., 2019). Moreover, the application of a biostimulant may be related to nutrient availability, and its direct role in plants by foliar application enhances the photosynthetic activity of enzymes and precursors of EOs, counting isoprenes, and phenylpropanes (Rehman et al., 2016). Rezaei-Chiyaneh et al. (2021) reported that biostimulant application in aromatic crops, *viz*., black cumin (*Nigella sativa* L.) and fenugreek (*Trigonella foenum-graecum* L.), enhanced EO quantity and quality. Additionally, EO profile varies according to climate, geographical features, and date of collection (Swamy and Sinniah, 2015). Accordingly chamazulene, bisabolone oxide, (α)-bisabolol, β-farnesene, bisabolol oxides A and B, and en-in-dicycloether were the major EO constituents of EO with biostimulant application under the lower altitude conditions of Iran (Ghasemi et al., 2016), while α-bisabolol oxide A, α-bisabolol oxide B, cis-β-farnesene, and bisabolone oxide were major constituents under the arid saline conditions of Egypt (Omer et al., 2013). The use of amino acid hydrolysates was also studied on maize and showed auxin-like activity and improved crop performance (Ugolini et al., 2014). The application of amino acids decreased chamazulene content and increased α-bisabolol oxide A content in German chamomile (El-Din and Abd El-Wahed, 2005). Other findings on amino acid application recorded higher α-bisabolol oxide B, α-bisabolol oxide A, and bisabolone oxide at higher doses, while at lower doses of application, (Z)-β-farnesene was higher (Omer et al., 2013). The present study can represent a facet for the accomplishment of sustainable means in German chamomile cultivation, particularly by taking into consideration the possible measures of reducing expenses on chemicals and increasing the opportunities for long-term sustainable plant nutrition by integrating chemical and sustainable sources of organic origin for crop production. The results validated that utilization of biostimulants directly increases the yield and influences the EO profile of German chamomile, which is particularly significant to farmers and straightforwardly indicates monetary benefits. Successively, from the customers' viewpoint, it is now more essential that the execution of this agronomic practice in the field offers their produce with an improved therapeutic and industrial perspective. The findings of the present study showed that German chamomile cultivation could be effectively initiated with the combination of P fertilization and biostimulant application under the acidic soil conditions of the western Himalayas. The hypothesis assumes that P and biostimulant application in acidic soils is fully justified considering German chamomile production and the economic concerns of growers.

## Conclusion

The present study aims to improve the availability of P to plants by applying a readily available source of P fertilizer in combination with foliar biostimulant applications for direct absorption by German chamomile plants. The results suggested that the combination of a synthetic P nutrient source and biostimulants can positively affect plant growth, flower yield, and EO composition. According to research, optimal utilization of P fertilizer and biostimulants improved the performance of German chamomile concerning flower production, biomass of flowers, and EO yield. The present research suggested that the utilization of P fertilizers and plant-based biostimulants is efficient for crop improvement in acidic soils, thus contributing to increased yield goal of growers. Considering the different reactions of P and biostimulants on agronomic and quality traits of German chamomile under stressed acidic conditions, it is recommended that these can be utilized in acidic soils of high rainfall regions. The lowest chamazulene and bisabolone oxide A content was obtained with the control, which means that when the availability of P is low, the yield attributes and chamazulene and bisabolone oxide A content will severely decrease. Similarly, the biostimulants also increased the content of chamazulene, α-bisabolol oxide A, (Z)-β-farnesene, and en-in-dicycloether in the EO of German chamomile. Detailed understanding of the effects of P and biostimulants will significantly provide benefits to various cropping systems and eventually lead to improved environmental steadiness by increasing crop yield and quality and reducing chemical inputs slowly and steadily for long-term benefits.

## Data Availability Statement

The original contributions presented in the study are included in the article/supplementary material, further inquiries can be directed to the corresponding author/s.

## Author Contributions

SR: data curation, methodology, visualization, validation, investigation, formal analysis, software, and writing—original draft and review and editing. RK: conceptualization, data curation, formal analysis, funding acquisition, investigation, methodology, project administration, resources, supervision, validation, and writing—review and editing. All authors contributed to the article and approved the submitted version.

## Conflict of Interest

The authors declare that the research was conducted in the absence of any commercial or financial relationships that could be construed as a potential conflict of interest.

## Publisher's Note

All claims expressed in this article are solely those of the authors and do not necessarily represent those of their affiliated organizations, or those of the publisher, the editors and the reviewers. Any product that may be evaluated in this article, or claim that may be made by its manufacturer, is not guaranteed or endorsed by the publisher.

## References

[B1] Abdelgawad K. F. Mhmoud A. A. Mohamed H. F. Y. (2020). Foliar spraying with some biostimulants improves growth, chemical constituents, and yield of head lettuce plant *Middle East*. J. Agric. Res. 7, 1268–1277.

[B2] Abdel-Mawgoud A. M. R. El-Greudy N. H. M. Helmy Y. I. Singer S. M. (2007). Responses of tomato plants to different rates of humic based fertilizer and NPK fertilization. J. Appl. Sci. Res. 3, 169–174.

[B3] Adams P. R. (2017). Identification of Essential Oil Components by Gas Chromatography/ Mass Spectroscopy, 4.1 ed. Suite A Carol Stream, IL: Allured Publishing Corporation, Gundersen Drive.

[B4] Amer A. Ghoneim M. Shoala T. Mohamed H. I. (2021). Comparative studies of eco-friendly compounds like humic acid, salicylic, and glycyrrhizic acids and their nanocomposites on French basil (*Ocimum basilicum* L. cv. Grand verde). Environ. Sci. Pollut. Res. 28, 47196–47212. 10.1007/s11356-021-14022-1 33886052

[B5] Andrzejewska J. Woropaj-Janczak M. (2014). German chamomile performance after stubble catch crops and response to nitrogen fertilization. Ind. Crops Prod. 62, 350–358. 10.1016/j.indcrop.2014.09.004

[B6] Anonymous (2020). Crop Weather Outlook. All India Coordinated Research Project on Agrometeorology (AICRPAM). Available online at: http://www.cropweatheroutlook.in/ (accessed October 1, 2020).

[B7] Balabanova D. A. Paunov M. Goltsev V. Cuypers A. Vangronsveld J. Vassilev A. (2016) Photosynthetic performance of the imidazolinone resistant sunflower exposed to single combined treatment by the herbicide imazamox an amino acid extract. Front. Plant Sci. 7, 1–10. 10.3389/fpls.2016.01559 PMC5078751 27826304

[B8] Burbott A. J. Loomis D. (1969). Evidence for metabolic turnover monoterpene in peppermint. Plant Physiol. 44, 173–179. 10.1104/pp.44.2.173 16657041 PMC396057

[B9] Can O. D. Ozkay U. D. H. D Demirci B. (2012). Psychopharmacological profile of Chamomile (Matricaria recutita L.) essential oil in mice. Phytomedicine. 19, 306–310. 10.1016/j.phymed.2011.10.001 22070986

[B10] Chaudhry A. N. Jilani G. Khan M. A. Iqbal T. (2009). Improved processing of poultry litter to reduce nitrate leaching and enhance its fertilizer quality. Asian J. Chem. 21, 4997–5003.

[B11] Chen Y. Shi X. Zhang L. Baskin J. M. Baskin C. C. Liu H.. (2019). Effects of increased precipitation on the life history of spring- and autumn-germinated plants of the cold desert annual *Erodium oxyrhynchum* (Geraniaceae). AoB Plants. 11, plz004. 10.1093/aobpla/plz004 30881621 PMC6410494

[B12] Cheng X. An S. Li B. Chen J. Lin G. Liu Y.. (2006). Summer rain pulse size and rainwater uptake by three dominant desert plants in a desertified grassland ecosystem in northwestern China. Plant Ecol. 184, 1–12. 10.1007/s11258-005-9047-6

[B13] Chimura M. Ikushima M. Miyazaki T. Kimura M. (1993). Effect of phosphorus on growth and concentration of mineral elements and essential oils of sweet basil leaves. Acta Horticulturae 396, 195–202. 10.17660/ActaHortic.1995.396.23

[B14] Chiyaneh S. F. Rezaei-Chiyaneh E. Amirnia R. Afshar R. K. Siddique K. H. M. (2022). Changes in the essential oil, fixed oil constituents, and phenolic compounds of ajowan and fenugreek in intercropping with pea affected by fertilizer sources. Ind. Crops Prod. 178, 1–11. 10.1016/j.indcrop.2022.114587

[B15] Chrysargyris A. Panayiotou C. Tzortzakis N. (2016). Nitrogen and phosphorus levels affected plant growth, essential oil composition and antioxidant status of lavender plant *Lavandula angustifolia* Mill. Ind. Crops Prod. 83, 577–586. 10.1016/j.indcrop.2015.12.067

[B16] Conselvan G. B. Fuentes D. Merchant A. Peggion C. Francioso O. Carletti P. (2018) Effects of humic substances indole-3-acetic acid on arabidopsis sugar amino acid metabolic profile. Plant Soil. 426, 17–32. 10.1007/s11104-018-3608-7

[B17] De Hita D. Fuentes M. Fernández V. Zamarreño A. M. Olaetxea M. García-Mina J. M. (2020). Discriminating the short-term action of root and foliar application of humic acids on plant growth: emerging role of jasmonic acid. Front. Plant Sci. 11, 1–14 10.3389/fpls.2020.00493 32411165 PMC7199506

[B18] Dehsheikh A. B. Sourestani M. M. Zolfaghari M. Enayatizamir N. (2020). Changes in soil microbial activity, essential oil quantity, and quality of Thai basil as response to biofertilizers and humic acid. J. Clean. Prod. 256, 1–10. 10.1016/j.jclepro.2020.120439

[B19] Drobek M. Frac M. Cybulska J. (2019). Plant biostimulants: importance of the quality and yield of horticultural crops and the improvement of plant tolerance to abiotic stress—A Review. Agronomy. 9, 1–18. 10.3390/agronomy9060335

[B20] El-Din K. M. G. Abd El-Wahed M. S. A. (2005). Effect of some amino acids on growth and essential oil content of chamomile plant. Int. J. Agric. Biol. 7, 1–5.7525047

[B21] El-Gohary A. E. Amer H. M. Salem S. H. Hussein M. S. (2019). Foliar application of selenium and humic acid changes yield, essential oil, and chemical composition of *Plectranthus amboinicus* (Lour.) plant and its antimicrobial effects. Egypt. Pharm. J. 18, 356–367. 10.4103/epj.epj_22_19

[B22] Emongor V. E. Chweya J. A. (1992). Effect of nitrogen and variety on essential-oil yield and composition from chamomile flowers. Trop. Agric. 9, 290–292.

[B23] Erbas S. Kucukyumuk Z. Baydar H. Erdal I. Sanli A. (2017). Effects of different phosphorus doses on nutrient concentrations as well as yield and quality characteristics of lavandin *Lavandula* × *intermedia* Emeric ex Loisel. var. Super. Turk. J. Field Crops. 22, 32–38. 10.17557/tjfc.301797

[B24] European Pharmacopoeia (2007). 6.0., Volume 1. Council of Europe, Strasbourg Cedex.

[B25] Fallah S. Mouguee S. Rostaei M. Adavi Z. Lorigooini Z. Shahbazi E. (2020). Productivity and essential oil quality of *Dracocephalum kotschyi* under organic and chemical fertilization conditions. J. Clean. Prod. 255, 1–11. 10.1016/j.jclepro.2020.120189

[B26] Fallah S. Rostaei M. Lorigooini Z. Surki A. A. (2018). Chemical compositions of essential oil and antioxidant activity of dragonhead (*Dracocephalum moldavica*) in sole crop and dragonhead-soybean (*Glycine max*) intercropping system under organic manure and chemical fertilizers. Ind. Crop. Prod. 115, 158–165. 10.1016/j.indcrop.2018.02.003

[B27] Gandomi A. Amiri B. Sharafzadeh S. Bazrafshan F. Hazrati S. (2021). The response of different fertilizer applications on chamomile production and their quality characteristics. Acta Sci. Pol. Hortorum Cultus. 20, 107–119. 10.24326/asphc.2021.2.11

[B28] Ghasemi M. Jelodar N. B. Modarresi M. Bagheri N. Jamali A. (2016). Increase of chamazulene and α-bisabolol contents of the essential oil of German chamomile (*Matricaria chamomila* L.) using salicylic acid treatments under normal and heat stress conditions. Foods. 5, 1–14. 10.3390/foods5030056 28231151 PMC5302395

[B29] Guo J.H. Liu X.J. Zhang Y. Shen J.L. Han W.X. Zhang W.F.. (2010). Significant acidification in major Chinese crop lands. Science. 327, 1–4. 10.9755/ejfa.2020.v32.i5.2099 20150447

[B30] Gurib-Fakim A. (2006). Medicinal plants: traditions of yesterday and drugs of tomorrow. Mol. Asp. Med. 27, 1–93. 10.1016/j.mam.2005.07.008 16105678

[B31] Jardin Du P. (2015). Plant biostimulants: definition, concept, main categories and regulation. Sci. Hortic. 196, 3–14. 10.1016/j.scienta.2015.09.021

[B32] Jeshni M. G. Mousavinik M. Khammari I. Rahimi M. (2015). The changes of yield and essential oil components of German chamomile (*Matricaria recutita* L.) under application of phosphorus and zinc fertilizers and drought stress conditions. J. Saudi Soc. Agric. Sci. 16, 60–65. 10.1016/j.jssas.2015.02.003

[B33] Jiang Y. Li Y. Zeng Q. Wei J. Yu H. (2017). The effect of soil pH on plant growth, leaf chlorophyll fluorescence and mineral element content of two blueberries. Acta Hortic. 1180, 269–276. 10.17660/ActaHortic.2017.1180.36

[B34] Jindo K. Canellas L. P. Albacete A. Figueiredo dos Santos L. Rocha R. L. F. Baia D. C. (2020) Review on interaction between humic substances plant hormones for phosphorous acquisition. Agronomy. 10, 1–18. 10.3390/agronomy10050640.

[B35] Johan P. D. Ahmed O. H. Omar L. Hasbullah N. A. (2021). Phosphorus transformation in soils following co-application of charcoal and wood ash. Agronomy. 11, 1–25. 10.3390/agronomy11102010

[B36] Kapoor R. Giri B. Mukerji K. G. (2004). Improved growth and essential oil yield and quality in *Foeniculum vulgare* mill on mycorrhizal inoculation supplemented with P-fertilizer. Bioresour. Technol. 93, 307–311. 10.1016/j.biortech.2003.10.028 15062827

[B37] Karami A. Khoush khoui M. Sefidkon F. (2006). Effects of nitrogen, phosphorus and potassium on yield and quantitative characteristics of cultivated and wild populations of German Chamomile (*Chamomilla recutita* L. Rauschert). Iran. J. Horticult. Sci. Technol. 7, 181–192.

[B38] Kocira S. Kocira A. Szmigielski M. Piecak A. Sagan A. Malaga-Tobola U. (2015). Effect of an amino acids-containing biostimulator on common bean crop. Przem. Chem. 94, 1732–1736. 10.15199/62.2015.10.16

[B39] Kumar S. Reager M. L. Pareek B. L. (2006). Yield components of mothbean (*Vigna aconitifolia* (Jacq.) Marechal) as influenced by phosphorus and bio fertilizer. Ann. Agric. Res. 27, 227–229.

[B40] Kwiatkowski C. A. Haliniarz M. Harasim E. Kołodziej B. Yakimovich A. (2020). Foliar applied biopreparations as a natural method to increase the productivity of garden thyme (*Thymus vulgaris* L.) and to improve the quality of herbal raw material. Acta Sci. Pol. Hortorum Cultus. 19, 107–118. 10.24326/asphc.2020.1.10

[B41] Lal R. K. (2008). Stability and genotypes × environment interactions in fennel. J. Herbs Spices Med. Plants. 13, 47–54. 10.1300/J044v13n03_05

[B42] Li X. Xiao J. He B. (2018). Higher absorbed solar radiation partly offset the negative effects of water stress on the photosynthesis of Amazon forests during the 2015 drought. Environ. Res. Lett. 13, 1–13. 10.1088/1748-9326/aab0b1

[B43] Lichtenthaler H.K. Buschmann C. (2001). Chlorophylls and carotenoids: measurement and characterisation by UV-VIS spectroscopy, in Current Protocols in Food Analytical Chemistry, eds Wrolstad R.E. Acree T.E. An H. Decker E.A. Penner M.H. Reid D.S. et al. (New York, NY: John Wiley and Sons), pp. F4.3.1–F4.3.8.

[B44] Ma Q. Longnecker N. Atkins C. (2001). Varying phosphorous supplied and development, growth and seed yield in narrow leafed lupin. Plant Soil. 239, 79–85. 10.1023/1014988219743

[B45] Ma Q. Wang X. Yuan W. Tang H. Luan M. (2021). The optimal concentration of KH_2_PO_4_ enhances nutrient uptake and flower production in rose plants *via* enhanced root growth. Agriculture. 11, 1–14. 10.3390/agriculture11121210

[B46] Mann C. Staba E. J. (2002). The chemistry, pharmacology and commercial formulations of chamomile. In: Craker LE Simon JE editors. Herbs, spices and medicinal plants- recent advances in botany, horticulture and pharmacology. USA: Haworth Press Inc.> 235–80.

[B47] Maurya K. R. (1989). Growth, yield and quality component in coriander genotypes. Indian J. Hortic. 46, 107–110.

[B48] Mazrou R. Ali E. F. Hassan S. Hassan F. A. S. (2021). A pivotal role of chitosan nanoparticles in enhancing the essential oil productivity and antioxidant capacity in *Matricaria chamomilla* L. Horticulturae. 7,574. 10.3390/horticulturae7120574

[B49] Meena R. L. Meena S. S. Mehta R. S. Meena R. D. (2015). Response of varying nitrogen and phosphorus levels on growth and yield of anise (*Pimpinella anisum* L.). Int. J. Seed Spices. 5, 83–87.

[B50] Metcalfe D. B. Meir P. Aragão L. E. O. C. Costa A. C. L. Braga A. P. Gonçalves P. H. L.. (2008). The effects of water availability on root growth and morphology in an Amazon rainforest. Plant Soil. 311, 189–199. 10.1007/s11104-008-9670-9

[B51] Mikhak A. Sohrabi A. Kassaee M. Z. Feizian M. (2017). Synthetic nanozeolite/nanohydroxyapatite as a phosphorus fertilizer for German chamomile (*Matricaria chamomilla* L.). Ind. Crops Prod. 95, 444–452. 10.1016/j.indcrop.2016.10.054

[B52] Mohamed Y. F. Y. (2020). Impact of some growth stimulants in cooperation with arbuscular mycorrhizal fungi on growth, productivity and chemical constituents of Dutch fennel plant Sci. J. Flowers Ornam. Plants. 7, 303–319. 10.21608/sjfop.2020.114567

[B53] Omer E. A. Said-Al Ahl H. A. H. Gendy A. G. E. Shaban K. A. Hussein M. S. (2013). Effect of amino acids application on production, volatile oil and chemical composition of chamomile cultivated in saline soil at Sinai. J. Appl. Sci. Res. . 9, 3006–3021.

[B54] Omidbeygi R. (1995). Approaches of Production and Processing of Medicinal Plants. Tehran: Behnashr Publication.

[B55] Orav A. Raal A. Arak E. (2010). Content and composition of the essential oil of *Chamomilla recutita* (L.) Rauschert from some European countries. Nat. Prod. Res. 24, 48–55. 10.1080/14786410802560690 20013472

[B56] Padilla F. M. Miranda J. D. Armas C. Pugnaire F. I. (2015). Effects of changes in rainfall amount and pattern on root dynamics in an arid shrubland. J. Arid Environ. 114, 49–53. 10.1016/j.jaridenv.2014.11.005

[B57] Purwanto B. H. Wulandari P. Sulistyaningsih E. Utami S. N. H. Handayani S. (2021). Improved corn yields when humic acid extracted from composted manure is applied to acid soils with phosphorus fertilizer. Appl. Environ. Soil Sci. 20, 1–12. 10.1155/2021/8838420

[B58] Ramezani S. Rezaei M. R. Sotoudehnia P. (2009). Improved growth, yield and essential oil content of basil grown under different levels of phosphorus sprays in the field. J. Appl. Biol. Sci. 3, 105–110.

[B59] Rathore S. Kumar R. (2021). Agronomic interventions affect the growth, yield, and essential oil composition of German chamomile (*Matricaria chamomilla* L.) in the western Himalaya. Ind. Crops Prod. 171, 1–11. 10.1016/j.indcrop.2021.113873

[B60] Rathore S. Mukhia S. Kapoor S. Bhatt V. Kumar R. Kumar R. (2022). Seasonal variability in essential oil composition and biological activity of *Rosmarinus officinalis* L. accessions in the western Himalaya. Sci. Rep. 12, 1–11. 10.1038/s41598-022-07298-x 35228638 PMC8885650

[B61] Rathore S. V. S. Dera D. K. Chand U. (1985). Studies of nitrogen nutrition through foliar spray of urea on the performance of African marigold. Udyarika 5, 37–40.

[B62] Rehman R. Hanif M. A. Mushtaq Z. Al-Sadi A. M. (2016). Biosynthesis of essential oils in aromatic plants: a review. Food Rev. Int. 32, 1–45. 10.1080/87559129.2015.1057841

[B63] Rezaei-Chiyaneh E. Battaglia M. L. Sadeghpour A. Shokrani F. Nasab A. D. M. Raza M. A.. (2021). Optimizing intercropping systems of black cumin (*Nigella sativa* L.) and fenugreek (*Trigonella foenum-graecum* L.) through inoculation with bacteria and mycorrhizal fungi. Adv. Sustain. Syst. 5, 1–14. 10.1002/adsu.202000269

[B64] Rioba N. B. Itulya F. M. Saidi M. Dudai N. Bernstein N. (2015). Effects of nitrogen, phosphorus and irrigation frequency on essential oil content and composition of sage (*Salvia officinalis* L.). J. Appl. Res. Med. Aromat. Plants. 2, 1–9. 10.1016/j.jarmap.2015.01.003

[B65] Rouphael Y. Spichal L. Panzarova K. Casa R. Colla G. (2018). High-Throughput Plant Phenotyping for Developing Novel Biostimulants: From Lab to Field or from Field to Lab? Front. Plant Sci. 9, 1–19. 10.3389/fpls.2018.01197 30154818 PMC6102389

[B66] Said-Al Ahl H. A. H. El Gendy A. G. Omer E. A. (2016). Humic acid and indole acetic acid affect yield and essential oil of dill grown under two different locations in Egypt. J. Pharm. Sci. Res. 8, 146–157.

[B67] Salehi A. Fallah S. Zitterl-Eglseer K. Kaul H. P. Abbasi Surki A. Mehdi B. (2019). Effect of organic fertilizers on antioxidant activity and bioactive compounds of fenugreek seeds in intercropped systems with buckwheat. Agronomy. 9, 1–16. 10.3390/agronomy9070367

[B68] Sangwan N. S. Farooqi F. S. Sangwan R,S. (2001). Regulation of essential oil production in plants. Plant Growth Regul. 34, 3–21. 10.1023/A:1013386921596

[B69] Sedibe M. M. Allemann J. (2012). Yield and quality response of rose geranium (*Pelargonium graveolens* L.) to sulphur and phosphorus application. S. Afr. J. Plant Soil. 29, 151–156. 10.1080/02571862.2012.744108

[B70] Sharafzadeh S. Alizadeh O. (2011). German and Roman Chamomile. J. Appl. Pharm. Sci. 1, 1–5.

[B71] Sofi A. Ebrahimi M. Shirmohammadi E. (2018). Effect of humic acid on germination, growth, and photosynthetic pigments of Medicago sativa L. under salt stress. Ecopersia 6, 21–30. 10.1001/1.23222700.2018.6.1.3.825996397

[B72] Song X. Razavi B. S. Ludwig B. Zamanian K. Zang H. Kuzyakov Y.. (2020). Combined biochar and nitrogen application stimulates enzyme activity and root plasticity. combined biochar and nitrogen application stimulates enzyme activity and root plasticity. Sci. Total Environ. 735, 139393. 10.1016/j.scitotenv.2020.139393 32492566

[B73] Sonmez C. (2018). Effect of phosphorus fertilizer on some yield components and quality of different anise (*Pimpinella anisum* L.) populations. Turkish J. Field Crop. 23, 100–106. 10.17557/tjfc.467468

[B74] Stein S.E. (2005). Mass Spectral Database and Software. Version 3.02. Gaithersburg, MD: National Institute of Standards and Technology (NIST).

[B75] Steward C. L. S. Lovett-Doust L. (2003). Effect of phosphorous treatment on growth and yield in the medicinal herbs *Calendula officinalis* L. Standart Pasific under hyproponic cultivation. Canadian J. Plant Sci. 83, 611–617. 10.4141/P02-132

[B76] Swamy M. K. Sinniah U. R. (2015). A comprehensive review on the phytochemical constituents and pharmacological activities of *Pogostemon cablin* Benth.: An aromatic medicinal plant of industrial importance. Molecules. 20, 8521–8547. 10.3390/molecules20058521 25985355 PMC6272783

[B77] Talaat I. M. Khattab H. I. Ahmed A. M. (2014) Changes in growth, hormones levels essential oil content of Ammi visnaga L. plants treated with some bioregulators. Saudi J. Biol. Sci. 21, 355–365. 10.1016/j.sjbs.2013.10.008 25183946 PMC4150225

[B78] Ubessi C. Tedesco S. B. da Silva C. B. D. Wagner R. Klein B. Andriolo J. L. A. (2021). Chemical composition of chamomile essential oil cultivated with homeopathy. J. Essent. Oil Res. 33, 342–350. 10.1080/10412905.2021.188551131029759

[B79] Ugolini L. Cinti S. Righetti L. Stefan A. Matteo R. D'Avino L.. (2014). Production of an enzymatic protein hydrolyzate from defatted sunflower seed meal for potential application as a plant biostimulant. Ind. Crops Prod. 75, 15–23. 10.1016/j.indcrop.2014.11.026

[B80] Upadhyay R. K. Singh V. R. Tewari S. K. (2016). New agro-technology to increase productivity of chamomile (*Matricaria chamomilla* L.). Ind.Crops Prod. 89, 10–13. 10.1016/j.indcrop.2016.04.072

[B81] Yang C. M. Wang M. C. Lu Y. F. Chang I. F. Chou C. H. (2004). Humic substances affect the activity of chlorophyllase. J. Chem. Ecol. 30, 1057–1065. 10.1023/B:JOEC.0000028467.82191.f9 15274448

[B82] Zavaleta-Mancera H. A. Lopez-Delgado H. Loza-Tavera H. Mora-Herrera M. Trevilla-Garcia C. Vargas-Suarez M.. (2007). Cytokinin promotes catalase and ascorbate peroxidase activities and preserves the chloroplast integrity during dark- senescence. J. Plant Physiol. 164, 1572–1582. 10.1016/j.jplph.2007.02.003 17485137

[B83] Zheng S. J. (2010). Crop production on acidic soils: overcoming aluminium toxicity and phosphorus deficiency. Ann. Bot. 106, 183–184. 10.1093/aob/mcq134 20570831 PMC2889811

[B84] Zouaoui N. Chenchouni H. Bouguerra A. Massouras T. Barkat M. (2020). Characterization of volatile organic compounds from six aromatic and medicinal plant species growing wild in North African drylands. NFS J. 18 19–28. 10.1016/j.nfs.2019.12.001

